# Risk reduction before surgery. The role of the primary care provider in preoperative smoking and alcohol cessation

**DOI:** 10.1186/1472-6963-10-121

**Published:** 2010-05-12

**Authors:** Hanne Tønnesen, Pernille Faurschou, Helge Ralov, Ditte Mølgaard-Nielsen, Grethe Thomas, Vibeke Backer

**Affiliations:** 1 WHO-CC, Bispebjerg University Hospital, Bispebjerg Bakke 23, Copenhagen, DK-2400 KBH NV, Denmark; 2 Department of Orthopaedic Surgery, Bispebjerg University Hospital, Bispebjerg Bakke 23, Copenhagen, DK-2400 KBH NV, Denmark; 3 Department of Surgical Gastroenterology, Bispebjerg University Hospital, Bispebjerg Bakke 23, Copenhagen, DK-2400 KBH NV, Denmark; 4 GP Clinic, Jernbane Allé 99,2, Vanløse, DK-2720, Denmark; 5 Emergency Medicine and Emergency Medical Services. The Capital Region of Denmark, Kongens Vænge 2, Hillerød, DK-3400, Denmark; 6 Department of Lung Diseases, Bispebjerg University Hospital, Bispebjerg Bakke 23, Copenhagen, DK-2400 KBH NV, Denmark

## Abstract

**Background:**

Daily smokers and hazardous drinkers are high-risk patients, developing 2-4 times more complications after surgery. Preoperative smoking and alcohol cessation for four to eight weeks prior to surgery halves this complication rate. The patients' preoperative contact with the surgical departments might be too brief for the hospital to initiate these programmes. Therefore, it was relevant to evaluate a new clinical practice which combined the general practitioner's (GP) referral to surgery with a referral to a smoking and alcohol intervention in the surgical pathway.

**Methods:**

The design was an exploratory prospective trial. The outcome measured was the number of patients referred to a preoperative smoking and alcohol cessation programme at the same time as being referred for elective surgery by their GP. The participants consisted of 72 high-risk patients who were referred for elective surgery by 47 local participating GPs.

The GPs, nurses, and specialists in internal medicine, prehabilitation and surgery developed new clinical practice guidelines based on the literature and interviews with 11 local GPs about the specific barriers for implementing a smoking and alcohol cessation programme. The role of the GP was to be the gatekeeper: identifying daily smokers and hazardous drinkers when referring them to surgery; handing out information on risk reduction; and referring those patients identified to a preoperative smoking and alcohol cessation programme. The role of the hospital was to contact these patients to initiate smoking and alcohol cessation at the hospital out-patient clinic for life-style intervention.

**Results:**

The GPs increased their referral to the smoking and alcohol cessation programme from 0% to 10% (7/72 patients) in the study period.

**Conclusion:**

The effect of the study was limited in integrating the efforts of primary care providers and hospital surgical departments in increasing the up-take of preoperative smoking and alcohol cessation programmes aimed at smokers and harmful drinkers referred for surgery. New strategies for cooperation between GPs and surgical departments are urgently needed.

**Trial registration:**

J.nr. 2005-54-1781 in Danish Data Protection Agency.

J.nr. 07 268136 in Scientific Ethical Committee for Copenhagen and Frederiksberg Municipalities.

## Background

Daily smoking and harmful alcohol intake increases the development of postoperative complications by two to four times [1-4]. The most frequent problems after surgery for smokers are wound and pulmonary complications; for harmful drinkers it is infections, bleeding episodes, cardiopulmonary insufficiency and death [1-4].

It is well documented that preoperative smoking and drinking cessation programmes of 4-8 weeks duration, significantly reduce the increased risk of complications after surgery [5-7]. Yet, short programmes have not been shown to have any effect on the surgical outcomes, probably because they are followed by poorer tobacco and alcohol cessation rates [8-10]. Recent studies considered the 4-8 weeks preoperative intervention to be cost-effective [11,12], and furthermore the patients were considered to be positive and motivated to undertake preoperative lifestyle changes in high quality programmes at the hospital [13].

Currently, preoperative smoking and alcohol cessation are recommended as standard operating procedures for both smokers and hazardous drinkers scheduled for elective surgery [1,2,14-17]. However, the preoperative contact with the hospital may often be too short for the patient to fulfil a preoperative lifestyle intervention programme of 4-8 weeks without postponing the date of surgery. Thus the involvement of the referring general practitioner (GP) becomes relevant.

It is well-known that the GP plays an important role in smoking and alcohol intervention programmes. However, several barriers have been identified which prevent GPs' engaging in lifestyle interventions, especially smoking cessation intervention [18-22] (Table 1); further local barriers may also exist.

**Table 1:** Major barriers identified from the literature for GPs' systematic engagement in a tobacco and alcohol intervention program, and the efforts to overcome those barriers in the present study

Identified Barriers	Present efforts to overcome the barriers
Fear of infringing the patient's right to self-determination [32]	Information regarding smoking and harmful drinking as objective risk factors for surgery and of the risk-reduction programmes (according to the patient folder) respecting the patient's right to self-determination on informed basis.

Missed the opportunity for promotion of medical benefit and protections from harm (i.e. GPs only engage with patients with smoking-related problems) [23,32,33]	Focus on the evidence of risk-reduction in relation to the current surgical illness

GP limited consultation to addressing patient's agendas relating to surgery [23,24,33,34]	Focus on the evidence of the high-risks of surgery for smokers and harmful drinkers. Systematic approach to identify and intervene

Harming the relationship with the patient [32]	Dissemination of knowledge that the majority of patients expect the GP and the hospital to deal with lifestyle. Use of the surgical illness as a window of opportunity to offer intervention

Not part of the job [23,31]	Only including engaged GPs, who volunteer to participate after informed consent. Focus on the GPs as key persons to initiate the risk reduction programmes in due time prior to surgery.

Too time-consuming [23,28]	The extra workload for the GP was less than 5 minutes per referred high-risk patient for surgery. The resulting increase of the reimbursement was 1/3 for the specific consultation

Lacking confidence and knowledge [25,27-29,34-36]	Simplified the information material, referring process, and guidelines which were to be handled by the GPs

Time not spent effectively due to few quitters [23,34,37]	Distribute knowledge about the high effectiveness of preoperative smoking and alcohol intervention (60-90% quitters)

Shortage of smoking cessation experts to whom the patient could be referred to [23,25,28,30]	Easy access by telephone-answering-machine to smoking cessation expertise, who took over the contact with the smokers and harmful drinkers once referred

Anticipating patient's lack of motivation and interest [26,28,29]	Distribute knowledge that the majority of patients expect the GP and the hospital to deal with lifestyle. Use of the surgical illness as a window of opportunity to offer intervention

Our hypothesis was that GPs engaged under attractive conditions would refer more high-risk patients to lifestyle intervention programmes prior to surgery. Our aim was to evaluate a new integrated clinical practice for daily smokers and hazardous drinkers admitted for elective surgery.

## Methods

### Design

The study was an exploratory prospective trial, evaluating "before and after" outcomes in a clinical setting.

We defined a high-risk patient as a daily smoker or a hazardous drinker consuming more than 14 drinks per week for women and 21 drinks per week for men; one drink containing 12 g. of ethanol [3,13].

### Ethical approval

This study was approved by the Quality Committee of GPs in Copenhagen and the quality management at our hospital. The study was also approved by the Danish Data Protection Agency and reported to the Scientific Ethical Committee System. It was not necessary to seek patient consent, since according to Danish Policy the intervention was aimed at the implementation of guidelines, involving only doctors, nurses, and the organisation.

### Development of integrated guidelines and material

**Figure 1:**
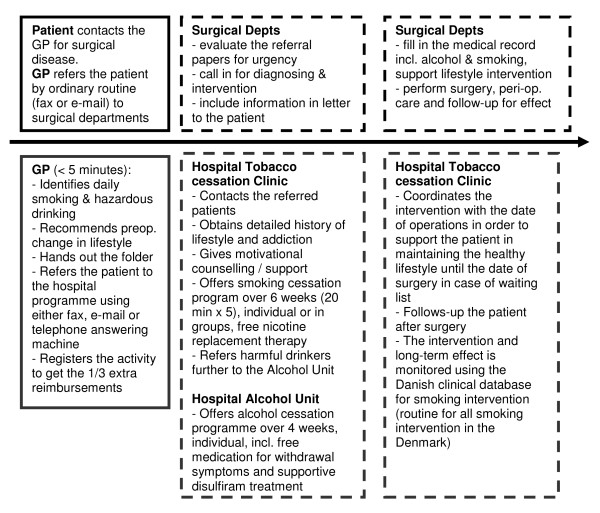
**Integrated preoperative guidelines for lifestyle intervention prior to surgery (the boxes above the arrow concern the pathway from GP to surgery, and the boxes under the arrow concern the integrated preoperative lifestyle intervention; closed boxes refer to the GPs and striated lines to the hospital activities)**.

A working group with key representatives from the local GPs, surgeons and nurses; Tobacco Cessation Clinic; Alcohol Unit; and Clinical Unit of Health Promotion was formed to develop the new integrated guideline for lifestyle intervention prior to surgery (Figure 1). This integrated guideline was based on the most recent evidence, which was exclusively gathered in hospital settings [1,4-7].

The working group decided how they would monitor the high-risk patient's preoperative route to surgery and give feedback (Additional file 1). They also developed a pack of materials for the GPs and surgeons. This pack consisted of: a folder describing how to identify high-risk patients; evidence of the effect of preoperative risk reduction programmes; recommendations for preoperative abstinence; and the procedure for referral to the hospital lifestyle intervention programme. Furthermore, the group developed a patient information folder (common to both the hospital and GPs). An information letter, the integrated guidelines and folder of material were sent to all 199 local GPs. The authors gave interviews to medical newspapers and web-portals about the project. The focus was on removing barriers to implementation by emphasising the benefits of the programme for the patient, and the simplicity of the referral procedure and the attractive reimbursement for the GPs, (The GPs received an additional 1/3 reimbursement per patient for identifying, informing and referral).

However, a few months after the start of the programme only two patients had been referred. In order to improve the referral rate, 11 of the first 12 GPs recruited to the project participated in a telephone interview regarding local barriers and facilitators for referral, as well as discussing further GP engagement and incentives. (Table 2) The resulting suggestions concerned patient-oriented efforts, such as an article in the local newspaper, free folders and posters in the clinic. The GP-oriented efforts included personal feedback every month; email reminders; a website; information at the doctor's desk; telephone calls; and involving the practice secretary. All the suggestions were subsequently taken up.

**Table 2:** Interview guide for the 11 GPs after inclusion of only 2 patients over 3 months

	Question	Response
1.	How many patients, aged 18 years and above, do you refer to the surgical departments at Bispebjerg Hospital annually?	6 GPs had 5-10 patients, 2 GPs had fewer, 2 had more, and 1 did not know.

2.	How many times have you handed out the information folder since the project started?	2 GPs had handed the information folder out on one occasion, 9 had not handed it out

3.	How many times have you informed a patient about this intervention programme?	1 GP had informed once, 1 twice and 9 had not informed any patients

4.	How did the patient react to this offer?	1 patient reacted with scepticism and 1 with a positive approach (both were included)

5.	Are you satisfied with the information level from the project group?	4 GPs said no and requested more information, especially reminders, 4 GPs said yes

6.	What do you think it would take to get more patients included in the study?	The GPs told that they often forgot the project. 5 GPs mentioned that it would be easier to remember the project, if it involved all surgical patients and hospitals in the Capital Region. 1 GP said that it would take 1 1/2 year for a GP to remember a new project.
		7 GPs wanted more reminders and 4 asked for more information
		*Patient-oriented suggestions: Article in the local newspaper, free folders and posters in the waiting room at the clinics.
		*GP-oriented suggestions: Personal feed-back and reminders by mail, e-mail, homepage, information card for doctor's desk, telephone call, and involving the practice secretary.
		1 GP wanted to use the fax for referral rather than the telephone answering machine.

### Outcome

The outcome parameter was the number of high-risk patients referred by GPs to the smoking and alcohol intervention programmes prior to surgery.

## Results

Prior to this study no high-risk patients had been referred to preoperative lifestyle intervention programmes by the local GPs.

72 high-risk patients, over a period of nine months, were referred for elective surgery at the departments of orthopaedic surgery and surgical gastroenterology by 47 local GPs who had volunteered to participate in the study. These 47 GPs had been recruited from 199 GPs based in the local community, by postal invitation and personal telephone calls. There were no exclusion criteria for recruiting local GPs.

**Figure 2:**
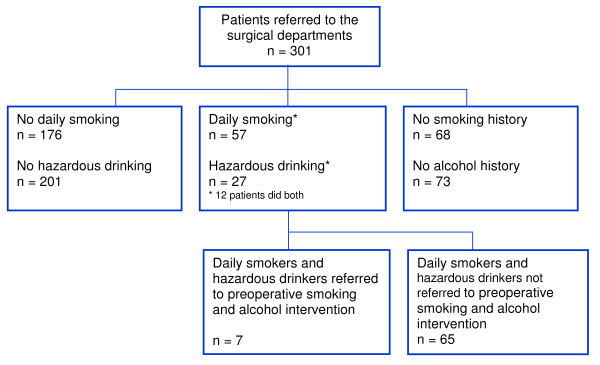
**Trial profile**. Patients referred by the 47 engaged GPs for elective surgery and preoperative smoking and alcohol intervention.

In total, 301 patients were admitted via the GPs throughout the study period for surgery. According to the preoperative surgical records, 57 of these 301 patients smoked daily (=19%) and 27 (9%) drank hazardously, including 12 patients who did both. The history of alcohol intake was unknown in 73 (=24%) patients, and the history of smoking was unknown in 68 (23%), (Figure 2). Only 7, of the identified 72, high-risk patients referred for elective surgery were also referred to preoperative smoking and alcohol intervention programmes. The 7 patients were referred by 7 different GPs (two of which belonged to the group of 11 interviewed GPs). The referrals were distributed over the total inclusion period. The GPs did not report any patients who were offered referral to the programme but refused.

## Discussion

In this study, we were only able to show a small increase, from zero to ten percent, in the frequency of patients directly referred by GPs to the surgical departments and the hospital's preoperative smoking and alcohol cessation programme. Though a ten percent increase in some situations is of importance, this low referral rate would not be acceptable in case of surgery from a patient safety view. This is because ninety percent of the high-risk patients that could benefit from a timely risk reduction are overlooked.

It was surprising that overcoming the barriers known to be related to GPs' assessment and counselling for tobacco cessation [23-37], did not facilitate the referral of high-risk patients in our study.

We discovered that in spite of a positive attitude among the GPs, as well as approval and support from their organisation, the referral rate was very low.

It might be speculated that this poor referral rate was due to patients refusing to be referred when offered this option by their GPs. However we discovered that this was not the case in the present study. We learnt in interviews with the GPs, that in daily practice, they forgot to play their role in risk reduction before surgery. In view of this the GPS asked for more information and reminders for both the patients and themselves. However, meeting these local wishes and suggestions did not result in an increased number of referred patients.

### Bias and limitations

Some of the elements in the integrated preoperative programme that had been identified in interviews or from previous studies as possible barriers have not yet been subjects for specific intervention research. They may therefore, theoretically, be of minor significance, which would reduce the effect of overcoming them. Another theoretical possibility could be that some of the efforts to overcome the barriers might have a negative effect and thereby neutralise a positive effect from other elements in the integrated programme. Furthermore, some barriers may be of greater importance in different countries; e.g. the simple referral model and easy access to smoking cessation experts has been shown to be effective among GPs in England [30], but not in the Netherlands [31]. The factors given in table 1 originated from Europe, North America and Australia; however, it is unclear whether the individual factors are specific to concrete contexts or health-care systems. The extra reimbursement for the GP reflected the "pay per service" or "quality-based reimbursement principle", which seemed promising in some studies, but not in others [38]. One other explanation for the poor referral rate could be that it is unusual tradition to have such integrated collaboration regarding risk reduction by changing lifestyle for surgical patients.

The tight time schedule imposed by the requirements of the integrated preoperative programme prior to surgery, could pose a significant challenge for GP's. In other situations the "window of intervention" for smoking and hazardous drinking may either be open longer or there is an opportunity to repeat the intervention programmes, for example in patients with COPD, diabetes, liver diseases etc.

Furthermore, the legal responsibility for the surgical pathway may play a role. According to the law in most countries the surgeon is the person responsible for having informed the patient sufficiently before surgery regarding the benefits and harms of that surgery. This is necessary to ensure that the patient has the correct basis for giving their informed consent to the operation.

### The clinical perspective

From a clinical point of view, integration of a preoperative smoking and alcohol cessation intervention in the surgical pathway is highly desirable. The effective preoperative lifestyle intervention programmes published hitherto either took place in the hospital out-patient clinics or in units in close relation to the surgical pathway and were given by experts [5-7].

In the long-term it would be relevant to establish a better tradition for collaboration between the primary care providers and the surgical departments. In the short-term it is, however, important to evaluate other strategies for risk reduction to offer the programmes in due time before surgery. Otherwise the consequences would be tremendous for the patient and for the healthcare system in general. In Denmark the annual costs related to the increased complications rate and prolonged hospital stay among harmful drinkers without preoperative lifestyle intervention programmes are approximately 30-50 Euros per capita [39]. The corresponding amount for smokers may be similar. The preoperative smoking intervention has already proven cost-effective in the immediate postoperative period [11,12].

The implementation of this programme may also arise from a political level, which may decide that all high-risk patients on waiting lists for elective operations should be offered a lifestyle intervention programme prior to surgery [40].

### The patient perspective

From a patient perspective, patients, relatives and patient organisations need improved information regarding the high-risk of surgery and the effectiveness of risk reduction programmes prior to surgery. Though smokers and hazardous drinkers undergoing surgery may never develop into a strong and demanding patient organisation, they may complain about misinformation or lost opportunities to improve their surgical outcomes in the future.

### The research perspective

This study gives rise to a hypothesis that overcoming identified barriers (table 1) are insufficient when implementing new integrated guidelines. It might be necessary to add new elements that can stimulate GPs to remember to follow the guidelines in their daily practice.

In addition, new research could change the emphasis from projects investigating barriers to exploration of fully implemented and integrated procedures in primary and secondary care. This may help to generate new strategies for implementation among surgical drinking and smoking patients.

## Conclusion

In conclusion, we did not succeed in using a programme integrating the efforts of the primary care providers and the hospital for risk reduction in the preoperative period aimed at smokers and harmful drinkers referred for surgery. Development, and evaluation, of new strategies for cooperation between primary care providers and surgical departments are urgently required in order to offer intervention programmes in a timely fashion before elective operations and to fulfil surgical obligations and responsibilities.

## Competing interests

HT, PF and HR have received funds for the present research as mentioned below. Bispebjerg Hospital, Copenhagen Health Services, GP Board of Quality Management, and Novartis might in some way, or to some degree, gain financially from the results. None of the funders took part in the design and conduct of the study; collection, management, analysis, interpretation of the data; preparation, review, or approval of the manuscript.

HT is a surgeon as well as director of the WHO Collaborating Centre for Evidence-Based Health in Hospitals; is working together with the National Board of Health and Danish Medical Association; is organising pre- and postgraduate education; and acts as peer reviewer on the subject for scientific journals.

PF is organising pre- and postgraduate education.

HR has no competing interest to be declared.

VB is associated professor, respiratory physician and chief of the respiratory research's unit as well as chief physician at the Tobacco Cession Unit.

DMN has no competing interest to be declared.

## Authors' contributions

HT has made substantial contributions to conception and design, acquisition of data, and analysis and interpretation of data. HT has been involved in revising the manuscript critically for important intellectual content and has given final approval of the version to be published. PF and HR have made substantial contributions to conception and design and analysis and interpretation of data. Both have been involved in revising the manuscript critically for important intellectual content and have given final approval of the version to be published. VB has made substantial contributions to conception and design. VB has been involved in revising the manuscript critically for important intellectual content and has given final approval of the version to be published. DMN and GT have made substantial contributions to acquisition of data. Both have been involved in revising the manuscript critically for important intellectual content and have given final approval of the version to be published.

## Pre-publication history

The pre-publication history for this paper can be accessed here:

http://www.biomedcentral.com/1472-6963/10/121/prepub

## Supplementary Material

Additional file 1 **Feedback**. Example of individual feedback to a GP. Click here for file
