# Supplementary material for: Risk reduction before surgery. The role of the primary care provider in preoperative smoking and alcohol cessation
Source: BMC Health Serv Res. 2010 May 12;10:121. doi: 10.1186/1472-6963-10-121 (PMC2882918; doi:10.1186/1472-6963-10-121)
Supplement: Additional file 1 — Feedback. Example of individual feedback to a GP. [file 1472-6963-10-121-S1.PDF]

Dear Dr. xxxx

Please, find below your personal feedback for the last month on the integrated preoperative care regarding ***Risk reduction for high-risk patients before surgery*.**

Referral from all the GPs in the local community during the last month of xxx

In total, all GPs referred 145 surgical patients to Bispebjerg Hospital in August (94 patients to Gastrointestinal Surgery, Department K and 51 patients to Orthopaedic Surgery, Department M); 31 patients (i.e. 21 %) were high-risk patients requiring preoperative smoking/drinking cessation programs:

Smoking history	*24 daily smokers**	*93 not daily smokers*	*28 no history*
Alcohol history	*12 hazardous drinkers**	*100 not hazardous drinkers*	*33 no history*

**5 patients were daily smokers with hazardous alcohol consumption.*

Referrals from GPs volunteering to use the integrated guidelines

35 patients were referred by the GPs participating in the project in the month of xxx.

Smoking history	*7 daily smokers**	*21 not daily smokers*	*7 no history*
Alcohol history	*5 hazardous drinkers**	*23 not hazardous drinkers*	*7 no history*

**2 patients were daily smokers with hazardous alcohol consumption.*

Personal feedback concerning your own referral

In this month you referred 3 patients to the two surgical departments at Bispebjerg Hospital; 2 were daily smokers, and 0 were hazardous drinkers. The 2 smokers were not referred to Tobacco Cessation Clinic.

_ *You have thereby given all your high-risk patients the opportunity to participate in the preoperative intervention programmes. Thank you very much.*

_ *You have had no high-risk patients and thus did not overlook the opportunity for risk reduction by preoperative intervention programmes. Thank you very much.*

X *You overlooked the opportunity for risk reduction among 2 of your patients by preoperative intervention programmes. You are welcome to contact us, in case you require any additional information.*

Thank you for your cooperation. We look forward to having more high-risk patients referred to the intervention programmes.

Yours sincerely

xxxx
